# Supplementary material for: Optimism/pessimism and associations with life event perceptions
Source: PLoS One. 2025 Apr 1;20(4):e0321128. doi: 10.1371/journal.pone.0321128 (PMC11960967; doi:10.1371/journal.pone.0321128)
Supplement: S1 Table — Note. Correlations r ≥ | .205 | are significant at p ≤ .05. Green cells are more positive; red cells are more negative; orange and yellow cells are in-between in terms of magnitude. (DOCX) [file pone.0321128.s001.docx]

| **S1 Table. Associations between Optimism and Pessimism and Event Characteristic Perceptions of Life Events** | | | | |
| --- | --- | --- | --- | --- |
| Life Event | Event Perception Characteristic | LOT-R Optimism | Optimism Subscale | Pessimism Subscale |
| Spent significant time in a different country | Challenge | -.063 | -.078 | .036 |
|  | Control | .005 | .107 | .093 |
|  | Emotional Significance | -.060 | -.193 | -.077 |
|  | Extraordinariness | .025 | -.075 | -.109 |
|  | Impact | .176 | .077 | **-.223** |
|  | Predictability | -.143 | -.083 | .161 |
|  | Social Status | -.036 | .119 | .171 |
|  | Valence | .062 | -.029 | -.133 |
|  | Worldview | .019 | -.095 | -.121 |
| Graduated college or university | Challenge | -.180 | -.049 | **.262** |
|  | Control | -.090 | .034 | .189 |
|  | Emotional Significance | .169 | .039 | **-.256** |
|  | Extraordinariness | -.039 | .070 | .140 |
|  | Impact | -.125 | -.195 | .026 |
|  | Predictability | -.032 | .090 | .146 |
|  | Social Status | -.052 | .117 | **.205** |
|  | Valence | .020 | -.109 | -.142 |
|  | Worldview | .070 | .025 | -.098 |
| Married | Challenge | -.150 | **-.224** | .061 |
|  | Control | -.128 | -.062 | .156 |
|  | Emotional Significance | .145 | .121 | -.139 |
|  | Extraordinariness | **-.300** | **-.346** | **.207** |
|  | Impact | .090 | .109 | -.056 |
|  | Predictability | -.072 | .047 | .156 |
|  | Social Status | -.096 | -.019 | .139 |
|  | Valence | .145 | .122 | -.136 |
|  | Worldview | .050 | .092 | -.011 |
| Moved to a new city or town at least 50 miles (80km) away | Challenge | .112 | .103 | -.092 |
|  | Control | **-.283** | -.186 | **.302** |
|  | Emotional Significance | -.053 | -.073 | .022 |
|  | Extraordinariness | -.063 | -.093 | .018 |
|  | Impact | .006 | -.022 | -.030 |
|  | Predictability | -.155 | -.010 | **.250** |
|  | Social Status | **-.208** | -.034 | **.319** |
|  | Valence | .015 | .082 | .050 |
|  | Worldview | .053 | .089 | -.007 |
| Made a new close friend | Challenge | **-.328** | -.140 | **.379** |
|  | Control | **-.277** | -.106 | **.312** |
|  | Emotional Significance | -.091 | -.061 | .093 |
|  | Extraordinariness | **-.260** | -.137 | **.279** |
|  | Impact | .015 | .122 | .081 |
|  | Predictability | .069 | .062 | -.028 |
|  | Social Status | -.174 | -.004 | **.270** |
|  | Valence | **.294** | .078 | **-.355** |
|  | Worldview | .192 | .096 | **-.211** |
| Became a parent | Challenge | .066 | .036 | -.080 |
|  | Control | .141 | **.213** | -.040 |
|  | Emotional Significance | .010 | .016 | -.006 |
|  | Extraordinariness | .126 | .101 | -.124 |
|  | Impact | .141 | .078 | -.179 |
|  | Predictability | -.119 | -.069 | .155 |
|  | Social Status | .195 | **.244** | -.103 |
|  | Valence | .046 | .072 | -.017 |
|  | Worldview | .169 | .109 | -.202 |
| Entered the workforce | Challenge | -.035 | -.139 | -.055 |
|  | Control | -.002 | .092 | .080 |
|  | Emotional Significance | .172 | **.271** | -.036 |
|  | Extraordinariness | .027 | .084 | .031 |
|  | Impact | -.007 | .070 | .070 |
|  | Predictability | -.143 | -.036 | .187 |
|  | Social Status | -.026 | .129 | .141 |
|  | Valence | .050 | -.101 | -.163 |
|  | Worldview | .191 | .195 | -.132 |
| Negative Events | | | | |
| Life Event | Event Perception Characteristic | LOT-R Optimism | Optimism Subscale | Pessimism Subscale |
| Victim of a serious crime | Challenge | **.228** | .073 | **-.312** |
|  | Control | -.125 | -.073 | .140 |
|  | Emotional Significance | .125 | .085 | -.130 |
|  | Extraordinariness | .185 | .061 | **-.251** |
|  | Impact | .046 | -.029 | -.103 |
|  | Predictability | -.062 | -.077 | .033 |
|  | Social Status | -.151 | -.040 | **.214** |
|  | Valence | -.175 | -.018 | **.273** |
|  | Worldview | .050 | .032 | -.055 |
| Experienced a natural disaster | Challenge | -.049 | -.122 | -.031 |
|  | Control | -.052 | .040 | .131 |
|  | Emotional Significance | -.095 | -.026 | .137 |
|  | Extraordinariness | -.025 | -.176 | -.124 |
|  | Impact | -.183 | -.140 | .181 |
|  | Predictability | .106 | -.010 | -.193 |
|  | Social Status | -.107 | .073 | **.259** |
|  | Valence | -.032 | .035 | .095 |
|  | Worldview | -.170 | -.094 | .203 |
| Divorced | Challenge | **.216** | .195 | -.203 |
|  | Control | -.016 | .011 | .036 |
|  | Emotional Significance | .163 | .157 | -.138 |
|  | Extraordinariness | .128 | .059 | -.163 |
|  | Impact | .028 | -.013 | -.060 |
|  | Predictability | .188 | **.261** | -.089 |
|  | Social Status | -.047 | -.052 | .033 |
|  | Valence | -.122 | -.134 | .093 |
|  | Worldview | **.243** | **.294** | -.162 |
| Had a falling out with a close friend | Challenge | -.083 | -.018 | .123 |
|  | Control | -.009 | .029 | .045 |
|  | Emotional Significance | -.090 | -.014 | .140 |
|  | Extraordinariness | -.022 | -.121 | -.075 |
|  | Impact | -.193 | -.137 | .203 |
|  | Predictability | -.051 | .005 | .104 |
|  | Social Status | **-.257** | -.155 | **.290** |
|  | Valence | .021 | .015 | -.022 |
|  | Worldview | .013 | .110 | .073 |
| Close family member (not parent) died | Challenge | .033 | .003 | -.054 |
|  | Control | -.004 | .125 | .126 |
|  | Emotional Significance | .129 | .084 | -.141 |
|  | Extraordinariness | .107 | .030 | -.155 |
|  | Impact | .023 | -.002 | -.041 |
|  | Predictability | .104 | .166 | -.019 |
|  | Social Status | .048 | .169 | .080 |
|  | Valence | -.011 | .066 | .083 |
|  | Worldview | .036 | .143 | .074 |
| Close family member jailed or imprisoned | Challenge | .120 | .029 | -.179 |
|  | Control | .086 | .087 | -.070 |
|  | Emotional Significance | .164 | .089 | -.199 |
|  | Extraordinariness | -.017 | -.142 | -.107 |
|  | Impact | .187 | .117 | **-.209** |
|  | Predictability | .155 | .055 | **-.219** |
|  | Social Status | .065 | -.001 | -.114 |
|  | Valence | -.141 | .010 | **.254** |
|  | Worldview | .166 | .078 | **-.212** |
| Fired from job | Challenge | -.054 | -.040 | .049 |
|  | Control | .046 | .097 | .008 |
|  | Emotional Significance | -.001 | .054 | .045 |
|  | Extraordinariness | -.046 | -.098 | -.011 |
|  | Impact | -.076 | -.025 | .108 |
|  | Predictability | .013 | .072 | .048 |
|  | Social Status | -.019 | .091 | .122 |
|  | Valence | .069 | .044 | -.071 |
|  | Worldview | .117 | .064 | -.150 |
| Close friend died | Challenge | **-.257** | **-.226** | **.247** |
|  | Control | -.025 | .024 | .069 |
|  | Emotional Significance | .037 | .036 | -.033 |
|  | Extraordinariness | -.034 | -.002 | .062 |
|  | Impact | -.113 | -.141 | .070 |
|  | Predictability | -.090 | -.048 | .116 |
|  | Social Status | -.110 | .006 | .202 |
|  | Valence | .017 | .036 | .003 |
|  | Worldview | .032 | .088 | .027 |
| Became seriously ill or injured | Challenge | .027 | .043 | -.012 |
|  | Control | -.105 | -.103 | .088 |
|  | Emotional Significance | .034 | .106 | .031 |
|  | Extraordinariness | .063 | -.006 | -.107 |
|  | Impact | .059 | .085 | -.028 |
|  | Predictability | .150 | .201 | -.080 |
|  | Social Status | -.126 | -.065 | .153 |
|  | Valence | .046 | .014 | -.062 |
|  | Worldview | -.073 | -.056 | .072 |
| Jailed or imprisoned | Challenge | .012 | -.065 | -.084 |
|  | Control | .173 | .162 | -.143 |
|  | Emotional Significance | .101 | .085 | -.092 |
|  | Extraordinariness | -.010 | -.032 | -.014 |
|  | Impact | .041 | -.012 | -.084 |
|  | Predictability | .201 | .100 | **-.253** |
|  | Social Status | .021 | .002 | -.036 |
|  | Valence | -.007 | .018 | .030 |
|  | Worldview | .178 | .206 | -.109 |
| Laid off from job | Challenge | .087 | .166 | .006 |
|  | Control | .039 | .150 | .067 |
|  | Emotional Significance | .096 | .165 | -.011 |
|  | Extraordinariness | .137 | .082 | -.147 |
|  | Impact | -.121 | -.057 | .143 |
|  | Predictability | .074 | .149 | .011 |
|  | Social Status | -.083 | -.028 | .107 |
|  | Valence | -.006 | .112 | .106 |
|  | Worldview | .117 | .148 | -.059 |
| Father or mother died | Challenge | .073 | .054 | -.077 |
|  | Control | **-.269** | **-.215** | **.259** |
|  | Emotional Significance | .181 | .196 | -.123 |
|  | Extraordinariness | -.022 | -.029 | .013 |
|  | Impact | .087 | .089 | -.066 |
|  | Predictability | -.169 | -.125 | .168 |
|  | Social Status | -.130 | .057 | **.291** |
|  | Valence | -.065 | -.049 | .068 |
|  | Worldview | .175 | .190 | -.120 |
| Romantic partner died | Challenge | .000 | -.112 | -.102 |
|  | Control | -.055 | -.059 | .040 |
|  | Emotional Significance | .041 | -.076 | -.143 |
|  | Extraordinariness | -.146 | -.197 | .069 |
|  | Impact | .176 | .012 | **-.295** |
|  | Predictability | .104 | .024 | -.158 |
|  | Social Status | -.048 | .014 | .099 |
|  | Valence | -.075 | .052 | .176 |
|  | Worldview | .087 | -.036 | -.187 |
| Note. Correlations *r* ≥ \|.205\| are significant at *p* ≤ .05. Green cells are more positive; red cells are more negative; orange and yellow cells are in-between in terms of magnitude | | | | |
