# Supplementary material for: Optimism/pessimism and associations with life event perceptions
Source: PLoS One. 2025 Apr 1;20(4):e0321128. doi: 10.1371/journal.pone.0321128 (PMC11960967; doi:10.1371/journal.pone.0321128)
Supplement: S1 Fig — Next two pages. Black marker: LOT-R optimism; red marker: optimism subscale; blue marker: pessimism subscale. (DOCX) [file pone.0321128.s002.docx]

**S1 Fig. Associations Between Optimism and Pessimism and Event Characteristic Perceptions of Positive Life Events.** Next two pages. Black marker: LOT-R optimism; red marker: optimism subscale; blue marker: pessimism subscale

*
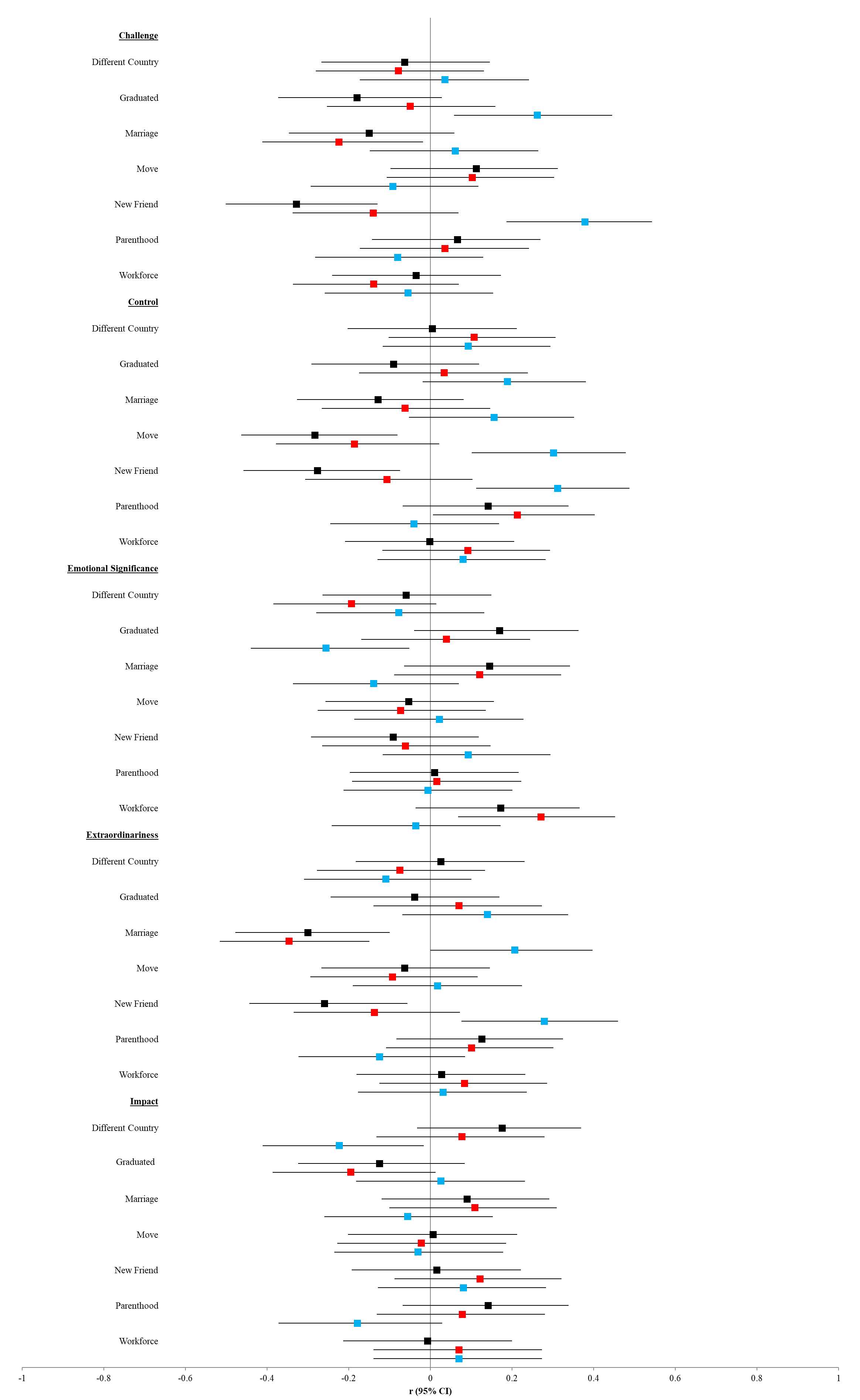
*

*
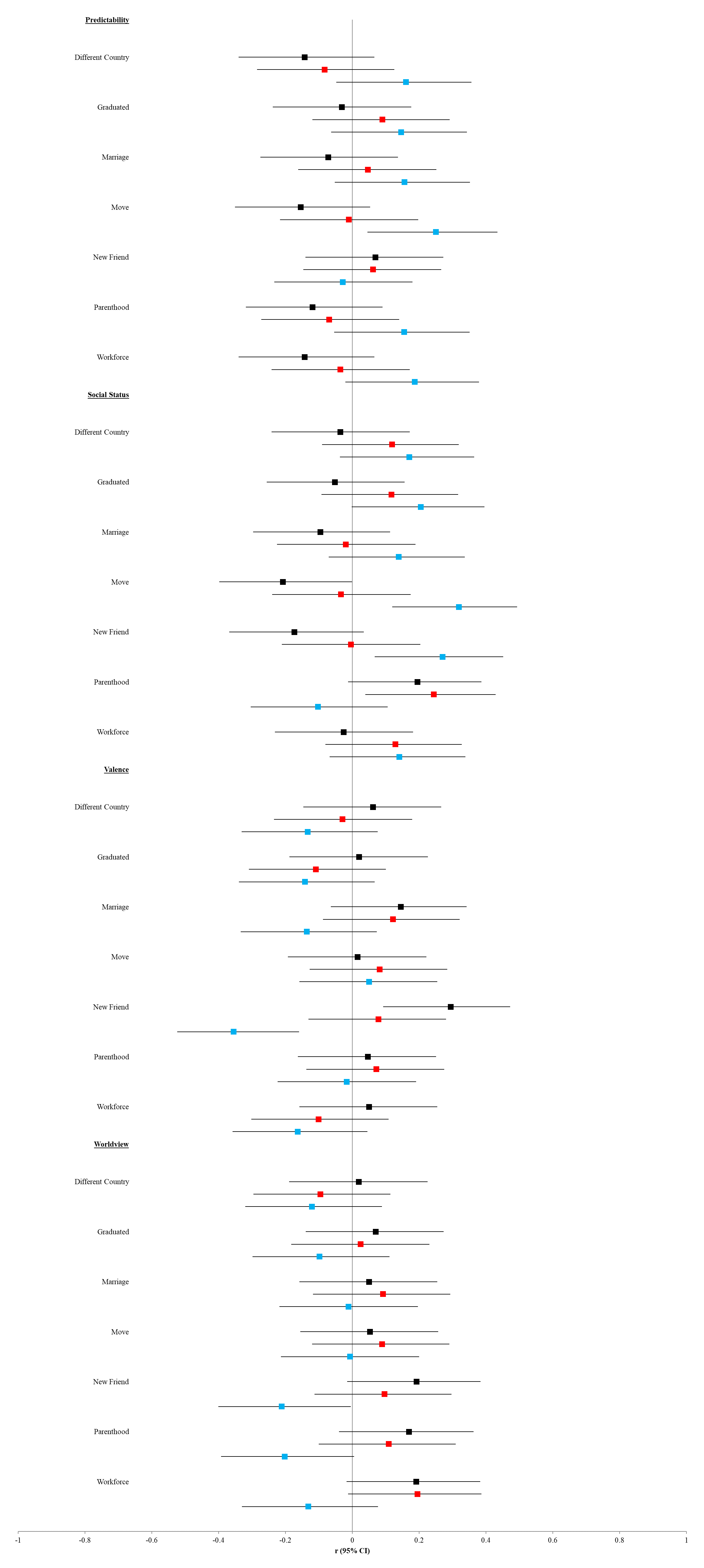
*
