# Supplementary material for: Optimism/pessimism and associations with life event perceptions
Source: PLoS One. 2025 Apr 1;20(4):e0321128. doi: 10.1371/journal.pone.0321128 (PMC11960967; doi:10.1371/journal.pone.0321128)

**S2 Fig. Associations between Optimism and Pessimism and Event Characteristic Perceptions of Negative Life Events.** Next five pages. Black marker: LOT-R optimism; red marker: optimism subscale; blue marker: pessimism subscale

*
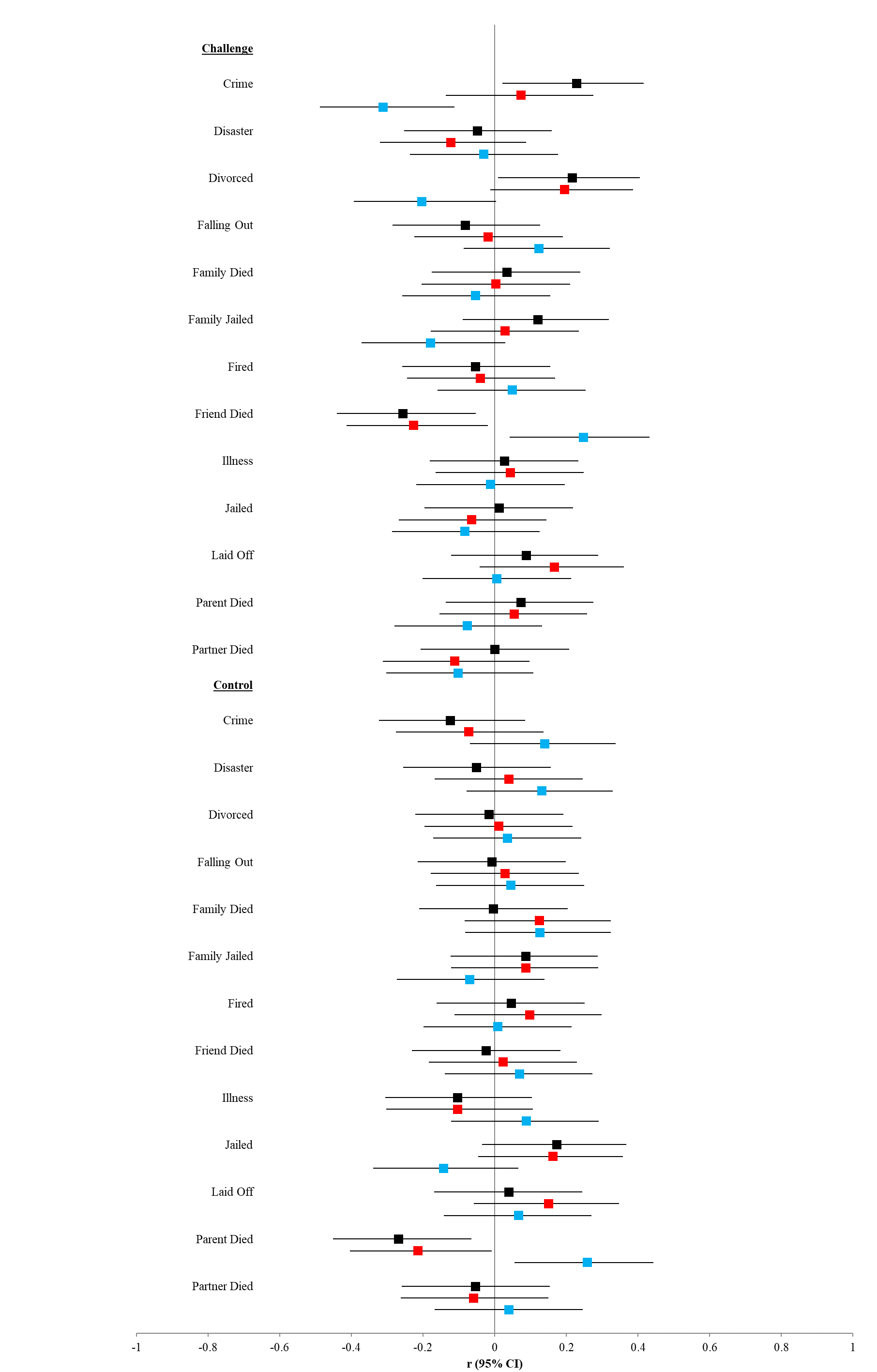
*


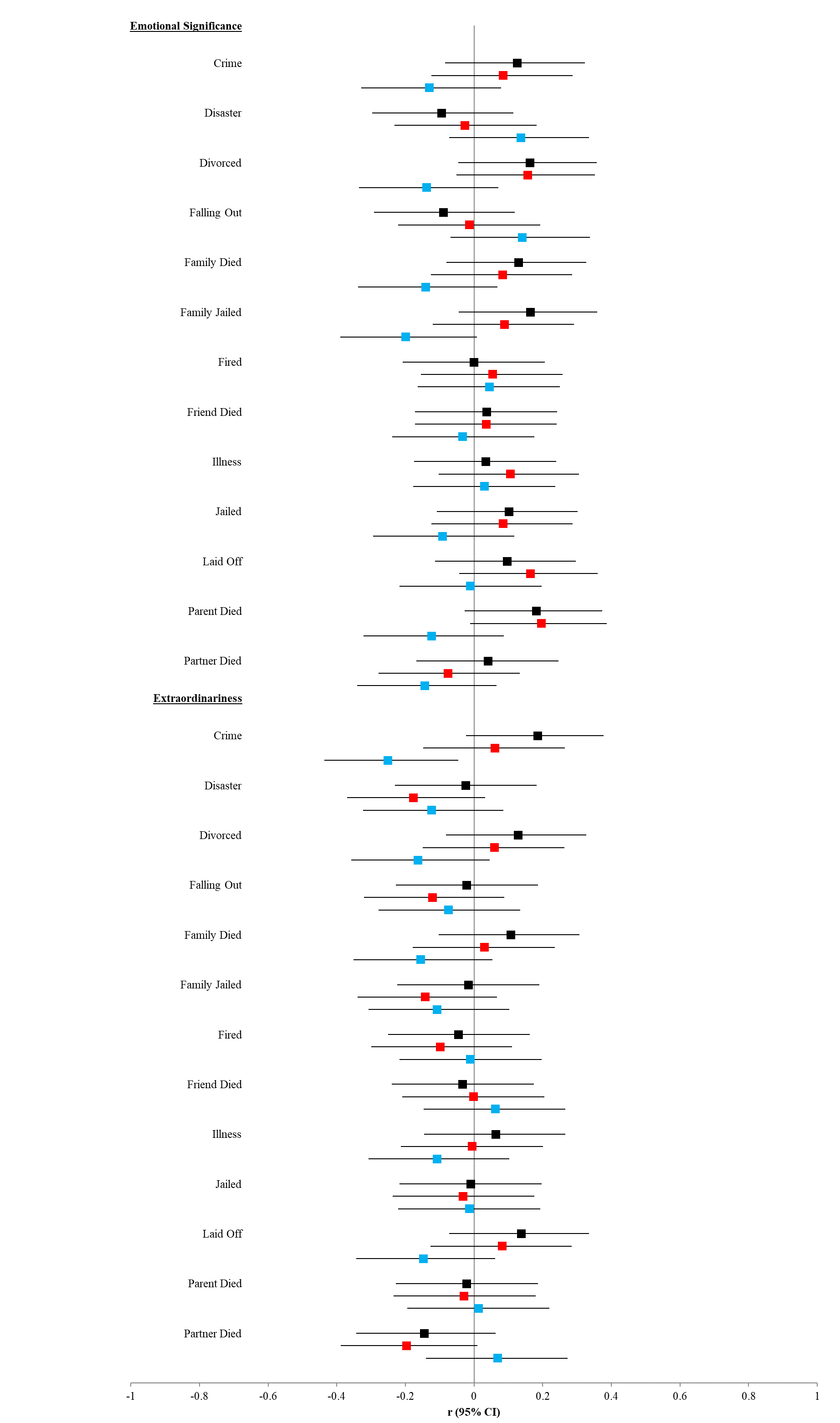


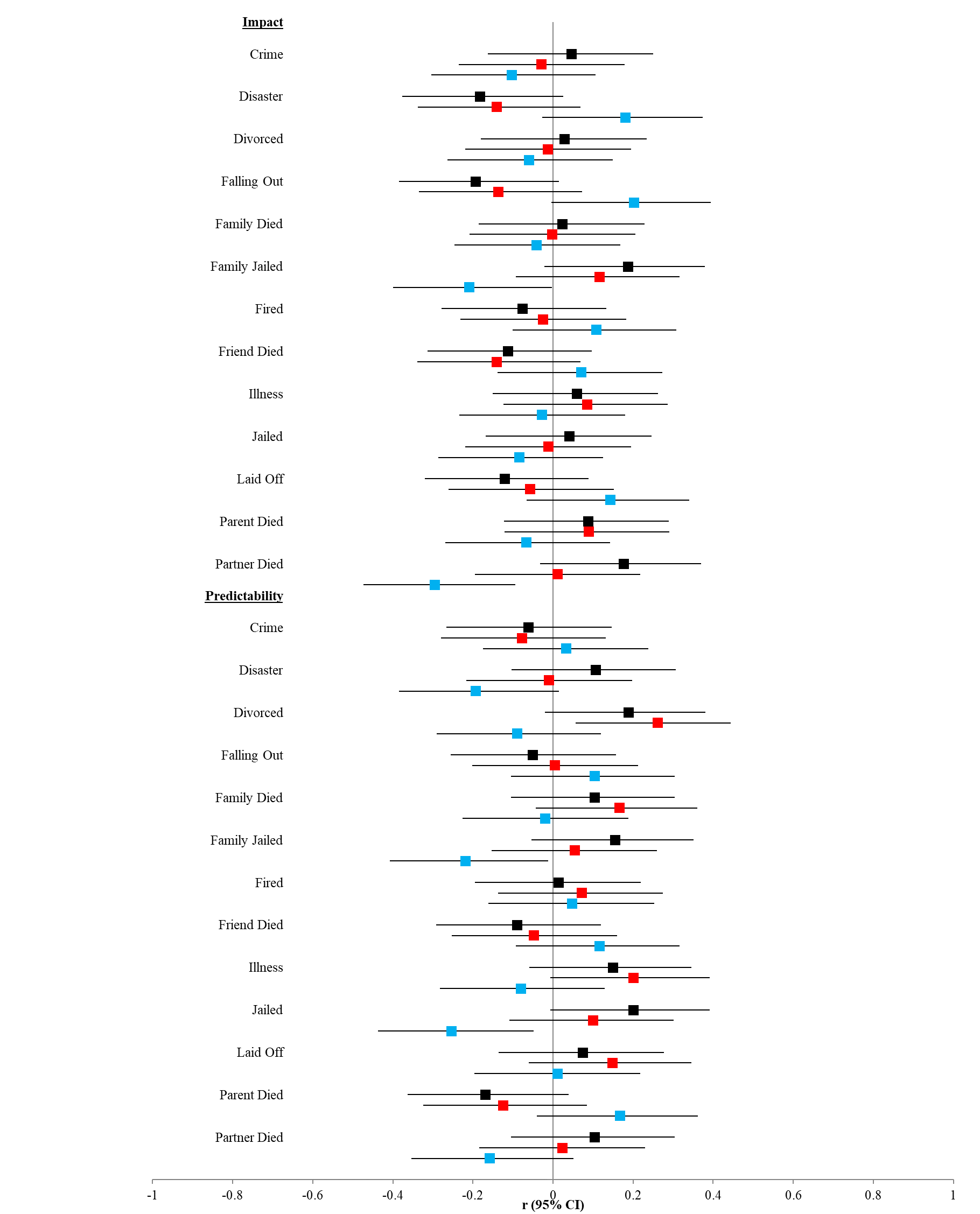


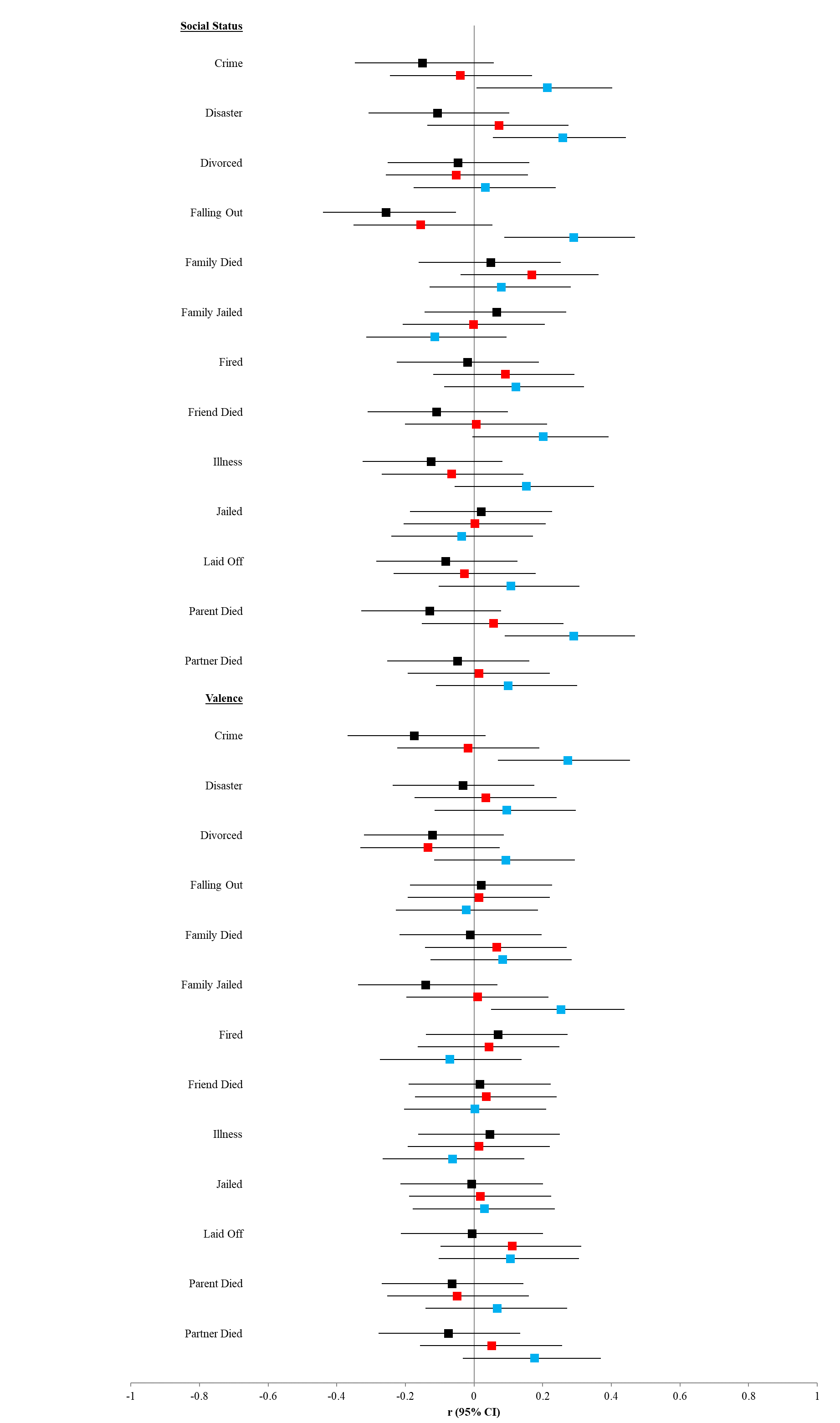


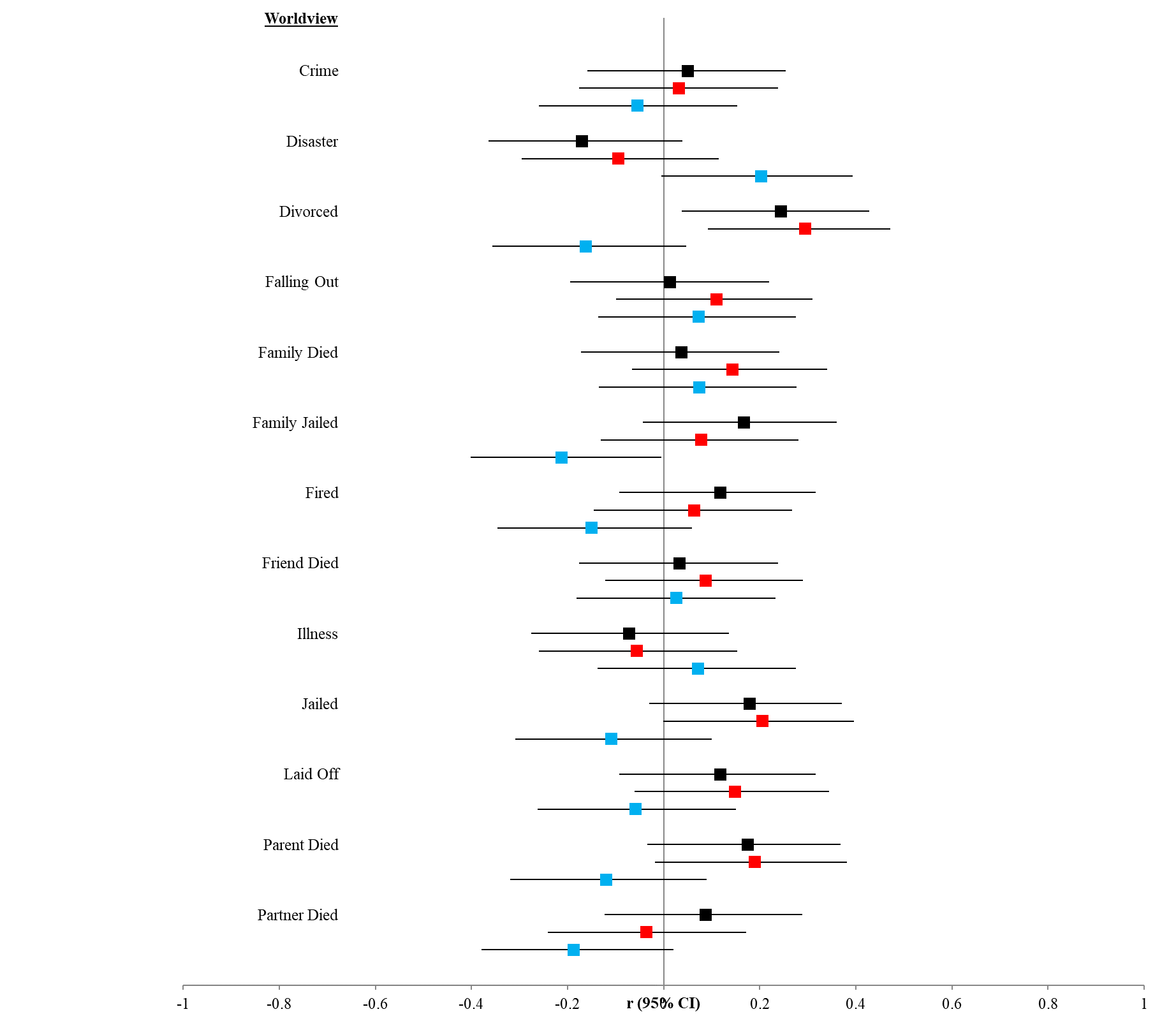

Supplement: S2 Fig — Next five pages. Black marker: LOT-R optimism; red marker: optimism subscale; blue marker: pessimism subscale. (DOCX) [file pone.0321128.s003.docx]
